# Marine Virus-Like Particles and Microbes: A Linear Interpretation

**DOI:** 10.3389/fmicb.2018.00358

**Published:** 2018-03-01

**Authors:** B. B. Cael, Michael C. G. Carlson, Christopher L. Follett, Michael J. Follows

**Affiliations:** ^1^Department of Earth, Atmospheric, and Planetary Sciences, Massachusetts Institute of Technology, Cambridge, MA, United States; ^2^Woods Hole Oceanographic Institution, Woods Hole, MA, United States; ^3^Faculty of Biology, Technion, Israel Institute of Technology, Haifa, Israel

**Keywords:** marine viruses, virus, virioplankton, viral abundance, virus-like particles, virus to microbe ratio, virus to bacterium ratio, virus-microbe relationship

Viruses are key players in ocean ecology and biogeochemistry, not only because of their functional roles but also partially due to their sheer abundance (Fuhrman, 1999; Wilhelm and Suttle, 1999). Because viruses cannot replicate without their hosts' machinery, their abundance is inextricably related to that of their (mostly microbial) hosts. The relationship between viral and microbial abundances is thus of great interest.

It is often assumed that the abundance of virus-like particles (*V*, mL^−1^) and microbial cells (*M*, mL^−1^) are approximately proportional. A rule-of-thumb “virus-to-microbe” ratio of 10 has been used (Thingstad, 2000) as measurements of *V* are frequently an order of magnitude larger than those of *M*. However, *V*/*M* is known to vary substantially (Maranger and Bird, 1995; Knowles et al., 2016; Wigington et al., 2016; Parikka et al., 2017).

Wigington et al. (2016) presented an alternative paradigm. They compiled 5,671 *V* and *M* measurements from 25 studies spanning diverse marine environments, an order of magnitude larger than any previous data compilation of its kind. They definitively showed that a power-law relationship *V* = α*M*^β^ is a better statistical model than a proportionality *V* = α*M*, in terms of the proportion of variance explained by each. They found β < 1 in most cases, meaning *V*/*M* decreases as *M* increases. If *V* does scale nonlinearly with *M* this has broad implications for the characterization of marine viruses' abundance and influence (Wigington et al., 2016), so it is important to be certain that a nonlinear model is superior to a linear description. It also raises questions as to how this nonlinear scaling emerges, and what determines α and β.

While several studies (Maranger and Bird, 1995; Danovaro et al., 2011; Knowles et al., 2016; Wigington et al., 2016) have tested the model *V* = α*M*^β^ by performing ordinary least-squares (OLS) regression on log-transformed *V* and *M* data, to our knowledge none have tested the linear model *V* = *aM*+*b*. Unlike *V* = α*M*, the linear model *V* = *aM*+*b* has the same number of parameters as the nonlinear model *V* = α*M*^β^, is consistent with a decreasing *V*/*M* as *M* increases, and is not a special case of the nonlinear model. We interpret *V* = *aM*+*b* to consider *V* as the sum of two pools—a pool of viruses whose abundance is proportional to microbial abundance (*aM*), and a background pool of other viruses and virus-like particles whose abundance is unrelated to microbial abundance (*b*). Because this model is simple and its parameters are comparatively straightforward to interpret and possibly predict, its ability to explain observations should be evaluated before concluding that *V* and *M* are nonlinearly related.

Here we evaluate the ability of the linear model *V* = *aM*+*b* to explain the relationship between *V* and *M* in the marine datasets considered by Wigington et al. (2016), Knowles et al. (2016), and Parikka et al. (2017). We show that the linear model's performance is never significantly different from that of the nonlinear model, and therefore that the linear model is a tenable description of the relationship between *V* and *M*.

We first reanalyzed the datasets considered in Wigington et al. (2016). They analyzed data from 25 studies, and also aggregated surface (*z* ≤ 100 m; *n* = 2, 921) and subsurface (*z* > 100 m; *n* = 2, 750) samples, totaling 27 datasets. We repeated their OLS regression of log_10_-transformed *V* and *M* data (see Supplementary Material for MATLAB R2017a code). We also fit the model *V* = *aM*+*b* after log-transformation:

$$\log_{10}V=\log_{10}(a10^{\log_{10}M}+b)$$

to the log-transformed data via nonlinear least-squares regression. Note that a linear relationship appears curved after log-transformation, asymptoting to *V* = *b* as *M* → 0 and to *V* = *aM* as *M* → ∞. We analyzed log-transformed data to be consistent with Wigington et al. (2016) and others (Maranger and Bird, 1995; Danovaro et al., 2011; Knowles et al., 2016), and because *M* and *V* span multiple orders of magnitude.

As the models have the same number of free parameters, we compared the two models by their coefficients of determination ($r_{l}^{2}$ and $r_{n}^{2}$ for the linear and nonlinear models respectively). We contend that if one model can be judged superior to the other based on these data alone, its superiority should be apparent even in the simplest statistical analyses.

Figure 1 shows $r_{l}^{2}$ vs. $r_{n}^{2}$ for all 27 datasets; neither model consistently outperforms the other and often their coefficients of determination are virtually the same^1^ (Table S1). The striking similarity between the models' performance suggests that one cannot discriminate between them based on these data alone.

**Figure 1:**
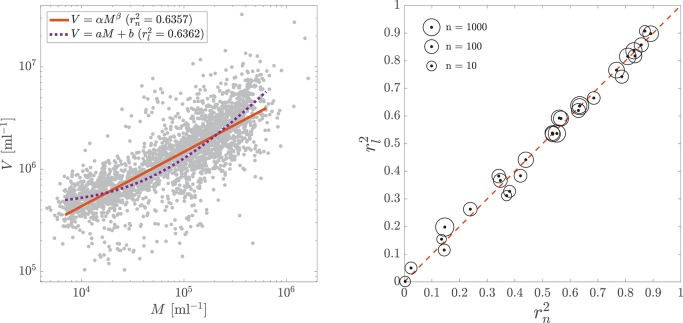
**(Left)** Example of linear model *V* = *aM*+*b* (dotted purple curve) compared to nonlinear model *V* = α*M*^β^ (solid red curve) for viral (*V*) and microbial (*M*) abundance data. Data are the subsurface samples (*z* > 100 m) from Wigington et al. (2016); compare to their Figure 3b. **(Right)** Coefficient of determination for linear model *V* = *aM*+*b* ($r_{l}^{2}$) vs. nonlinear model *V* = α*M*^β^ ($r_{n}^{2}$) for the 29 datasets considered in the text. Dashed red line corresponds to $r_{l}^{2}=r_{n}^{2}$. Scatterpoints are sized by the logarithm of the number of samples in each dataset. For each point, the difference between it and the red dashed line is not statistically significant at the 90% confidence level, as estimated by the bootstrap analysis described in the text.

To quantify the similarity of $r_{l}^{2}$ and $r_{n}^{2}$, we estimated their confidence intervals with a bootstrap analysis (Efron, 1979). For each of the 27 datasets, we generated 10,000 replicate datasets via resampling with replacement, repeated the above analysis on each replicate, and estimated the 90% confidence intervals of $r_{l}^{2}$ and $r_{n}^{2}$ for each dataset by the 5 and 95th percentiles of their bootstrap distributions (Table S1). For all 27 datasets the 90% confidence intervals for $r_{n}^{2}$ and $r_{l}^{2}$ overlap, indicating that the difference between the two is not statistically significant, even at the nonconservative 90% confidence level.

Knowles et al. (2016) and Parikka et al. (2017) also compiled *V* and *M* data from different environments, including marine environments. We repeated the above analyses on the marine data from both studies, and found that they corroborate our findings above. For the “Pelagic Marine” data from Parikka et al. (2017) (*n* = 221) we found $r_{l}^{2}=0.59$ and $r_{n}^{2}=0.57$ with overlapping 90% confidence intervals; for the “Deep Ocean” data^2^ from Knowles et al. (2016) (*n* = 18) we found $r_{l}^{2}=0.91$ and $r_{n}^{2}=0.87$ with overlapping 90% confidence intervals (Table S1).

Two earlier studies further corroborate that statistically a linear model is a tenable explanation of the *V*-*M* relationship. Maranger and Bird (1995; their Figure 2) (*n* = 149), and Danovaro et al. (2011; their Figure 1a) [*n* = 631, including the data from (Maranger and Bird, 1995)] also performed OLS regression of aggregated and log-transformed marine *V* and *M* data. Both studies estimated β≈1 – Maranger and Bird (1995) found β = 0.93, and Danovaro et al. (2011) found β = 1.03—though neither study's data were available to reanalyse and uncertainties on their estimates for β were not reported. The closeness of β to 1 is consistent with a linear relationship; nonlinearity requires β ≠ 1.

Thus, we conclude that the linear model *V* = *aM*+*b* and the nonlinear model *V* = α*M*^β^ fit this series of datasets of marine viral and microbial abundance equivalently well. This raises the questions of how each model should be interpreted as a description of the relationship between viral and microbial abundances.

The linear model can be interpreted as decomposing virus-like particle abundance into two terms: *V* = *aM*+*b* = *V*_*a*_+*V*_*b*_. In this interpretation, the first term *V*_*a*_ is the abundance of viruses that infect organisms included in *M*, and it is assumed that *V*_*a*_ ∝ *M* as captured by the parameter *a*. In marine systems, *M* is dominated by the abundance of prokaryotes, thus *V*_*a*_ can be considered to generally represent the abundance of bacteriophage (Suttle, 2007). *a* can then be considered a characteristic virus-to-microbe ratio, and can be understood in the context of dynamical models. For example, consider arguably the simplest model for virus-microbe dynamics (Lotka, 1920; Volterra, 1928; Lauro et al., 2011; Yau et al., 2011),

$$\frac{dV}{dt}=\;\gamma \varphi VM-\lambda V,\;\frac{dM}{dt}=\;\mu M-\varphi VM$$

where (γ, φ [mL s^−1^], λ [s^−1^], μ [s^−1^]) represent burst size, virus-microbe interactions, viral decay, and microbial growth respectively (Record et al., 2016). At steady state, this model predicts *V*/*M* = γμ/λ. Predictions for *a* can therefore be derived from such models. In the above model, *a* is the product of the viral burst size, the host growth rate, and the viral decay timescale—though most of these are poorly parameterized in environmental populations. Across the 29 datasets we considered, our estimates of *a* ranged from 0 to 92, with a median of 8.2 (Table S1). This large variation is similar to that of α and β (Table S1; Knowles et al., 2016; Wigington et al., 2016), and is also consistent with the large range in virus-to-microbe ratios observed across marine environments (Parikka et al., 2017). In a majority of cases *a* was within a factor of two of the “rule of thumb” virus-to-microbe ratio of 10.

The second term *V*_*b*_ is an additional “background pool” of virus-like particles, whose abundance is assumed to be unrelated to microbial abundance. Different types of particles other than infective bacteriophage may be present in this background pool, which could plausibly be variable and non-negligible. Virus-like particles are operationally defined as particles <0.2 μm in size containing nucleic acids; their abundance is measured by treating samples with a fluorescent nucleic acid stain, then counting fluorescing particles with epifluorescence microscopy (Noble and Fuhrman, 1998; Patel et al., 2007). Several non-viral sources are known to be captured by these methods whose contribution to total counts can be large, such as free nucleic acids (Bettarel et al., 2000), DNA-containing extracellular vesicles that are secreted by numerous marine microbes (Soler et al., 2015; Biller et al., 2017), gene-transfer agents (Biers et al., 2008), and decomposing viral material (Wommack et al., 1996). Additionally, while the relationship between microbes and viruses is generally thought to be dominated by prokaryotes and bacteriophage, eukaryote-infecting viruses (Nagasaki and Bratbak, 2010), zooplankton-infecting viruses (Fischer et al., 2010), and virophage (La Scola et al., 2008) may also contribute to *V* without their corresponding hosts contributing to *M*. Finally, virus-like particles and microbes have overlapping sizes and counting each population has a significant degree of subjectivity. Therefore, many different kinds of particles might be subsumed into *V*_*b*_; this background pool could change across environmental gradients (Biller et al., 2017) and with host types in ways unrelated to microbial density. Predictions for *b* could be made depending on which combination of these particle types *b* is assumed to represent. Across the 29 datasets we considered, our estimates of *b* also ranged substantially, from 0 to 6 × 10^7^ mL^−1^, with a median of 6 × 10^5^ mL^−1^. Biller et al. (2014) reported vesicle abundances of 3 × 10^5^ and 6 × 10^6^ mL^−1^ for coastal surface water and Sargasso Sea samples; interestingly, a majority of our estimates for *b* were between these two values (Table S1).

Interpreting the nonlinear model is less straightforward. Knowles et al. (2016) developed a theory of viruses switching from lytic to lysogenic lifestyles to explain the sublinear scaling relationships they observed, but their theory has been disputed (Weitz et al., 2017) and broadly speaking the relationships between viral and microbial abundances they find tend to be weak or have β≈1 (not indicative of nonlinearity); see their Figures 1a, 2. Empirical sublinear relationships are ubiquitous in biology (Hatton et al., 2015), but the underpinnings of the metabolic theory commonly invoked to explain these relationships have also been disputed (Dodds et al., 2001). It is unclear how the nonlinear model would account for virus-like particles other than bacteriophage, or how α and β would be interpreted and predicted.

These analyses highlight the need for alternative methods to quantify viruses in the environment relative to their hosts. Measurements from marine environments with extremely low microbial densities (because the two models diverge for low values of *M*), novel experiments with model systems, or other types of data that improve upon the indiscriminant measure of virus-like particles may help answer this question (Baran et al., 2018), and may also be useful for making accurate estimates of *b*. For now, we contest that a linear model remains plausible.

## Author contributions

BC conceived the research, performed the analyses, and wrote the paper. MC, CF, and MF assisted in the writing process and revised the manuscript.

### Conflict of interest statement

The authors declare that the research was conducted in the absence of any commercial or financial relationships that could be construed as a potential conflict of interest.

## Footnotes

^1^The large fluctuations evident in *V* compared to the range of *M* helps explain the models' equivocal performance and can yield correlations between *V* and *V*/*M* (Parikka et al., 2017).

^2^These are compiled from Muck et al. (2014); the other marine dataset considered by Knowles et al. (2016) is taken from Parsons et al. (2012) and is included in the Wigington et al. (2016) dataset and therefore in our analyses above.
